# Temperature-Driven Twin Structure Formation and Electronic Structure of Epitaxially Grown Mg_3_Sb_2_ Films on Mismatched Substrates

**DOI:** 10.3390/nano12244429

**Published:** 2022-12-12

**Authors:** Sen Xie, Yujie Ouyang, Wei Liu, Fan Yan, Jiangfan Luo, Xianda Li, Ziyu Wang, Yong Liu, Xinfeng Tang

**Affiliations:** 1 State Key Laboratory of Advanced Technology for Materials Synthesis and Processing, Wuhan University of Technology, Wuhan 430070, China; 2 International School of Materials Science and Engineering, Wuhan University of Technology, Wuhan 430070, China; 3 The Institute of Technological Sciences, Wuhan University, Wuhan 430072, China; 4 School of Physics and Technology and The Key Laboratory of Artificial Micro- and Nano-Structures of Ministry of Education, Wuhan University, Wuhan 430072, China

**Keywords:** Mg_3_Sb_2_ films, molecular beam epitaxy, strain relaxation, twin structure, electronic structure

## Abstract

Mg_3_Sb_2_-based compounds are one type of important room-temperature thermoelectric materials and the appropriate candidate of type-II nodal line semimetals. In Mg_3_Sb_2_-based films, compelling research topics such as dimensionality reduction and topological states rely on the controllable preparation of films with high crystallinity, which remains a big challenge. In this work, high quality Mg_3_Sb_2_ films are successfully grown on mismatched substrates of sapphire (000*l*), while the temperature-driven twin structure evolution and characteristics of the electronic structure are revealed in the as-grown Mg_3_Sb_2_ films by in situ and ex situ measurements. The transition of layer-to-island growth of Mg_3_Sb_2_ films is kinetically controlled by increasing the substrate temperature (*T*_sub_), which is accompanied with the rational manipulation of twin structure and epitaxial strains. Twin-free structure could be acquired in the Mg_3_Sb_2_ film grown at a low *T*_sub_ of 573 K, while the formation of twin structure is significantly promoted by elevating the *T*_sub_ and annealing, in close relation to the processes of strain relaxation and enhanced mass transfer. Measurements of scanning tunneling spectroscopy (STS) and angle-resolved photoemission spectroscopy (ARPES) elucidate the intrinsic p-type conduction of Mg_3_Sb_2_ films and a bulk band gap of ~0.89 eV, and a prominent Fermi level downshift of ~0.2 eV could be achieved by controlling the film growth parameters. As elucidated in this work, the effective manipulation of the epitaxial strains, twin structure and Fermi level is instructive and beneficial for the further exploration and optimization of thermoelectric and topological properties of Mg_3_Sb_2_-based films.

## 1. Introduction

Mg_3_Sb_2_-based compounds have attracted widespread attention in research areas of thermoelectrics and condensed matter physics due to their exceptional thermoelectric performances and novel topological properties. From the aspect of structure-property correlations, Mg_3_Sb_2_ is one type of Zintl compounds with phonon-glass and electron-crystal (PGEC) characteristics [1,2,3,4], beneficial for its thermoelectric performance. In the past decade, a variety of strategies have been utilized to boost the thermoelectric properties of Mg_3_Sb_2_, such as tuning carrier density with dopants, band engineering [5,6], forming solid solutions [7,8,9], and interface engineering [6,10,11,12], but research has seemed to hit a bottleneck. According to the latest theoretical calculations, a leap in the performance of Mg_3_Sb_2_ requires innovative strategies based on epitaxial technology [13,14,15,16]. Zhang et al. have proposed that, the crystal field energy splitting of valence bands could be modulated by substrate-induced epitaxial strains in Mg_3_Sb_2_ epi-films, bringing about the convergence of valence bands and the optimized *zT* of 0.7 [15]. In addition, based on Boltzmann transport calculations, Shan et al. have reported that the mono-layer Mg_3_Sb_2_ would be endowed with strong electrical properties and a low lattice thermal conductivity due to the beneficial dimensionality reduction, resulting in a very high *zT* of 2.5 at elevated temperatures [13]. Furthermore, in the process of exploring new topological systems, Mg_3_Sb_2_ was regarded as a suitable candidate possessing novel type-II nodal-line topological states, through appropriately modulating the spin-orbital coupling (SOC) strength of Mg_3_(Sb,Bi)_2_ solid solutions [17]. Zhou et al. have successfully synthesized high quality Mg_3_Bi_2_ films by the molecular beam epitaxy (MBE) technique, and have characterized the surface resonance bands by angle-resolved photoelectron spectroscopy (ARPES) [18]. However, the subtle control of the Bi/Sb ratio and the films’ crystallinity remained as the big challenge in Mg_3_(Sb,Bi)_2_ [17,19]. There are three obstacles regarding the epitaxial growth of Mg_3_Sb_2_-based films. (1) Due to the high saturation vapor pressure and chemical activity of the Mg, it is necessary to identify a series of substrates with matched lattice parameters that are chemically inert with Mg, as well as to effectively control the Mg content [20,21,22]. (2) To get desired epi-films with high crystallinity and with excellent compositional control, the optimal growth window for the growth of Mg_3_Sb_2_-based films should be thoroughly and carefully explored. (3) Epitaxial strains, twin structure and dislocations are usually discovered in epitaxial films under nanometer scale, which could largely influence the functional performances of materials [23,24]. Hence, it is necessary to clarify the tuning mechanisms of these structures in the epitaxially grown Mg_3_Sb_2_ films. 

In this work, the influences of MBE processing parameters on the phase structure and crystallinity of binary Mg_3_Sb_2_ films were systematically investigated, while the ARPES band structure was illustrated in the films grown under various MBE parameters. The transition of layer-to-island growth mode with increasing the film thickness, and its relation with epitaxial strains, were revealed in our Mg_3_Sb_2_ films. Moreover, the obvious twin structure in Mg_3_Sb_2_ films could be effectively manipulated by the substrate temperature (*T*_sub_), and hence, by the mass transfer process and epitaxial strains. Furthermore, the Fermi level (*E*_F_) showed a substantial shift of ~0.2 eV towards the conduction band as the Mg/Sb ratio (R) increased greatly, likely due to the suppression of Mg vacancies. This work clearly elucidated the rich nanostructure evolution during the epitaxial growth of Mg_3_Sb_2_ films, and lays the foundation for exploring novel band structures of Mg_3_Sb_2_-based materials through strain engineering or forming heterostructures.

## 2. Materials and Methods

Mg_3_Sb_2_ films with the highest crystallinity were mainly grown on Al_2_O_3_ (000*l*) substrates in a commercial Molecular Beam Epitaxy (MBE) system (Octoplus 300, Dr. Eberl MBE-Komponenten GmbH, Germany), while the growth of Mg_3_Sb_2_ films was also carried out on HOPG, BaF_2_ (111) and Ge (111) substrates. The standard RCA cleaning method (omitting the HF etching step) is used to remove the inorganic and organic impurities on the surface of Al_2_O_3_ (000*l*) substrates. High purity Mg (99.9%) and Sb (99.999%) were evaporated with the nominal Mg/Sn ratio R of 4:1~16/1 and at the Sb flux of 0.03 Å/s. The main experimental results were obtained from Mg_3_Sb_2_ films grown under R = 4:1, if not explicitly stated. The *T*_sub_ is selected as 573 K, 673 K and 773 K in this study. Two types of growth processes were selected: the one-step growth process and the growth and annealing process (annealed at 773 K after growth). The film growth process was monitored in situ by reflection high energy electron diffraction (RHEED). The crystal structure was examined using high-resolution x-ray diffraction (XRD, Smart Lab, Rigaku, Japan), including the *θ*-2*θ* scan, rocking curve and pole figure. The thickness and surface morphology of Mg_3_Sb_2_ films were characterized by an atomic force microscope (AFM, Dimension FastScan, Bruker, Germany). The valence band structure of Mg3Sb2 films was measured at 10 K in an angle resolved photoemission spectroscopy (ARPES) apparatus that is connected to the MBE chamber by a UHV transfer line. The analyser (Scienta Omicron DA-30L, Sweden) with an energy resolution of 7 meV and an angular resolution of 0.2°, the He-I monochromatic light source (Fermion Instruments, Shanghai) and the six-axis sample manipulator equipped a He closed-cycle helium refrigerator (Fermion Instruments, Shanghai) were utilized in the measurements. Scanning tunneling microscopy (STM) and scanning tunneling spectroscopy (STS) measurements were carried out in a UHV and low-temperature STM (LT-STM-AFM-N, CreaTec Fisher & Co. GmbH, Germany) operated at 77 K, which was connected to the MBE chamber by an UHV transfer line.

Prior to the film growth, the Mg_3_Sb_2_ layer with thickness of several nanometers was deposited on the Al_2_O_3_ (000*l*) substrate at 773 K, followed by a subsequent desorption at 1023 K until the substrate’s RHEED patterns were recovered. We noted that the introduction of this passivation process is beneficial for the epitaxial growth of Mg_3_Sb_2_ films with improved crystallinity, as shown in Figure S1. Such a process is broadly applicable for lattice-mismatched epitaxial growth, including WSe_2_ and other TMDCs [25]. 

## 3. Results

### 3.1. Lattice-Mismatched Epitaxial Growth of Mg_3_Sb_2_

**Figure 1:**
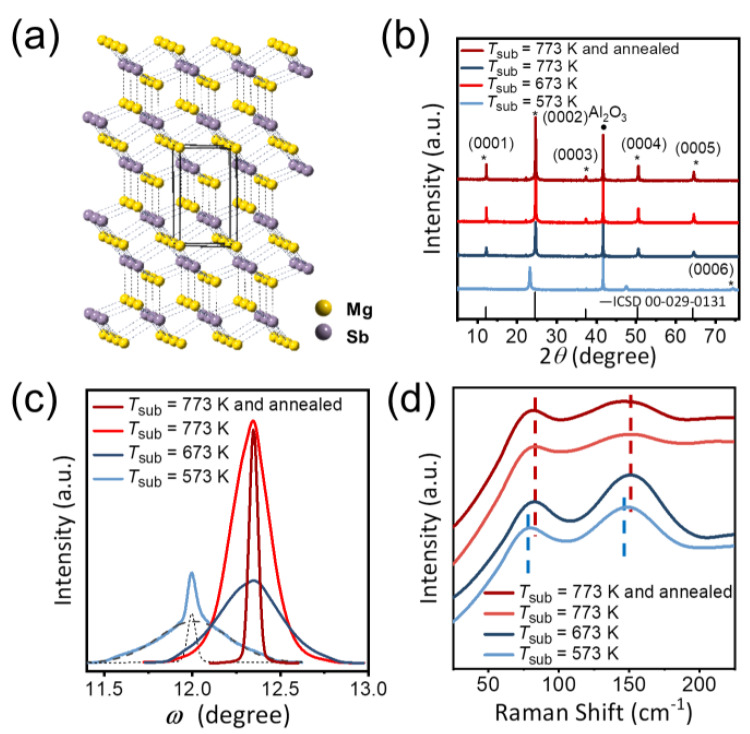
(**a**) Crystal structure of Mg_3_Sb_2_ drawn based on the tetragonal structure (space group R-3m, *a* = *b* = 4.55 Å, *c* = 7.23 Å), with comparable interlayer and intralayer bonds; (**b**) *θ-*2*θ* XRD patterns for the Mg_3_Sb_2_ films grown on Al_2_O_3_ (000*l*) substrates under different thermal processes. The Mg_3_Sb_2_ film grown at 573 K is submitted to a 5.4% tensile strain along the *c*-axis; (**c**) Rocking curves around the (0002) reflection of the grown Mg_3_Sb_2_ films in (**b**). The film grown and in situ annealed at 773 K exhibits the highest crystallinity and the lowest dislocation density, with FWHM = 0.12°. (**d**) The Raman spectra of the Mg_3_Sb_2_ films; the positions of the Raman peaks are marked with dotted lines.

Figure 1a shows the crystal structure of *α*-Mg_3_Sb_2_ (space group: $P\bar{3}m1$, No. 164). Mg_3_Sb_2_ compounds can be regarded as a special case of Zintl phase CaAl_2_Si_2_ (AB_2_X_2_) in which A and B are occupied by Mg1 (interlayer) and Mg2 (intralayer) atoms [26]. Since both the intralayer Mg2-Sb and interlayer Mg1-Sb are weak chemical bonds based on Bader charge analysis, Mg_3_Sb_2_ exhibits a nearly isotropic 3D bonding network and pseudo-layered feature [26]. This structural feature has been corroborated by recent discoveries, such as the nearly isotropic lattice thermal expansion [26], and the slightly higher compression ratio along the out-of-plane direction than that along the in-plane direction [27,28]. These findings highlight the unique chemical bonding characteristics of Mg_3_Sb_2_, distinct from the van der Waals (vdW) layered materials with weak interlayer interactions [29] and the 3D materials featuring strong anisotropic chemical bonds [30]. As a result, the pseudo-layered crystal structure endows Mg_3_Sb_2_ with exceptional tolerance of epitaxially growing on substrates without strict lattice-matching requirements. As shown in Figure 1b,c and Figure S2, the Mg_3_Sb_2_ films with strong (000*l*) orientation can be grown on lattice-mismatched substrates of Al_2_O_3_ (000*l*), cleaved HOPG, Ge(111) and BaF_2_ (111) substrates with in-plane lattice misfit of 4.6%, 8.1%, 12%, and −3.8%, respectively. According to the collected RHEED patterns, the Mg_3_Sb_2_ film grown on Al_2_O_3_ (000*l*) substrate possesses the highest crystalline quality, benefiting from the aforementioned passivation process and good anti-Mg corrosion resistance of the Al_2_O_3_ substrate. 

The crystalline structure of Mg_3_Sb_2_ films grown on Al_2_O_3_ (000*l*) substrates was investigated by high resolution XRD measurements, as illustrated in Figure 1b,c. Except for the peaks at ~42° and 21° originating from the Al_2_O_3_ substrate, the XRD (*θ*-2*θ* scan) peaks of films grown under different thermal processes can all be indexed to (000*l*) patterns of Mg_3_Sb_2_, confirming a strict *c*-axis orientation. Meanwhile, increasing *T*_sub_ combined with the annealing process could drastically reduce the full-width-half-maximum (FWHM) of the rocking curve from 0.95° to 0.11°, implying the prominent improvement of crystalline quality. The film grown and annealed at *T*_sub_ = 773 K has the narrowest rocking curve FWHM and the highest crystallinity among all films. In contrast, the film directly grown at *T*_sub_ = 573 K possesses the worst crystalline quality, and the diffraction peaks of this film shift obviously towards lower angles, as shown in Figure 1b. This result indicates that the film directly grown at *T*_sub_ = 573 K suffers from a tensile strain along the *c*-axis (*ε*_⊥_) with the lattice parameter expanding by 5.4% (*c* = 7.62 Å as compared to *c* = 7.23 Å in the pristine Mg_3_Sb_2_). Figure 1d displays the corresponding Raman spectra of the aforementioned Mg_3_Sb_2_ films grown under different thermal processes. All the films present two characteristic Raman active peaks at the range of 50–200 cm^−1^, similar to the recent result in Mg_3_Sb_2_ based bulks [31]. An obvious Raman redshift is observed in the strained film grown at *T*_sub_ = 573 K, while the frequencies of Raman spectra in the other films are nearly identical. The Raman peak shift is regarded as the indicator of strain state, in which the Raman peak position shifting to higher (or lower) frequencies reflects compressive (or tensile) strain being induced in the material [32]. Therefore, the Raman redshift verifies an increased bond length in the strained Mg_3_Sb_2_ film grown at *T*_sub_ = 573 K, consistent with the *c*-axis expansion of the Mg_3_Sb_2_ structure detected from XRD measurements. 

### 3.2. Growth Mode and Epitaxial Strain Control 

**Figure 2:**
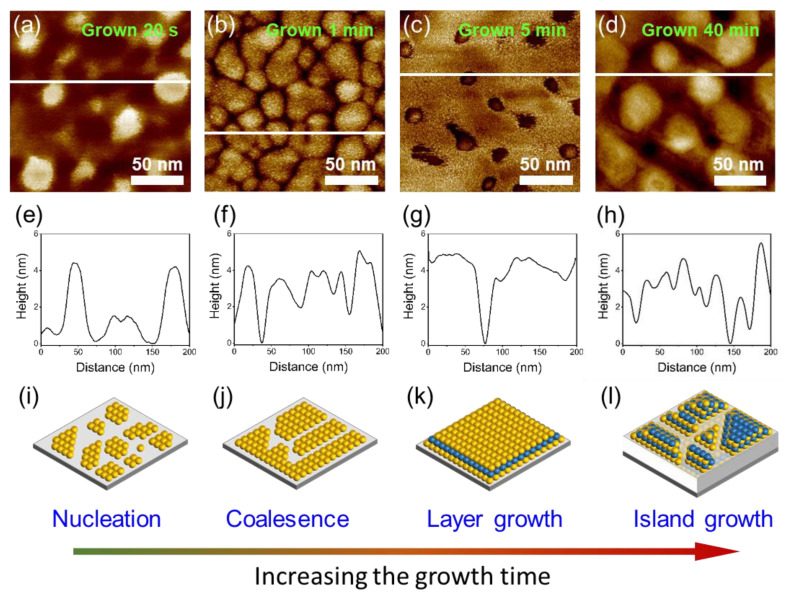
Surface morphologies and the film growth mode for Mg_3_Sb_2_ films grown on Al_2_O_3_ substrates. (**a**–**d**) AFM images at different stages of the film growth for the films grown at *T*_sub_ = 773 K; (**e**–**h**) The corresponding height profiles along the white lines in (**a**–**d**); (**i**–**l**) The schematic illustration of the film growth mode that fits the characteristic of the Stranski–Krastanov (SK) mode.

The growth of Mg_3_Sb_2_ films on Al_2_O_3_ (000*l*) substrate is classified as the Stranski–Krastanov (SK) mode featured with the layer-plus-island growth evolution during the film deposition, which is experimentally elucidated by AFM in Figure 2. During the initial growth period, the multipoint nucleation spontaneously forms at *t* ≈ 30 s with a nucleus size of 2~5 nm in height and 20~40 nm in diameter. The crystal nuclei grow quickly and coalesce into a flat Mg_3_Sb_2_ slab when the film is grown for 1~15 min. As the film thickness further increases, 3D island grains form on top of the initial flat Mg_3_Sb_2_ slab, implying a rough surface morphology. The SK growth mode implies the interaction between the Mg_3_Sb_2_ layer and Al_2_O_3_ substrate being distinctly stronger than that between Mg_3_Sb_2_ layers. The strong interaction introduces a driving force favoring for forming flat Mg_3_Sb_2_ layer during the initial growth. With increasing the film thickness of Mg_3_Sb_2_, the strong interaction between Mg_3_Sb_2_ and Al_2_O_3_ decays rapidly, and the island growth dominates the subsequent growth process. The SK growth characteristic is intimately related to the relaxation of strain in the case of mismatched epitaxy and to the control of the twin structure, which will be clarified in our Mg_3_Sb_2_ films, as noted below. 

**Figure 3:**
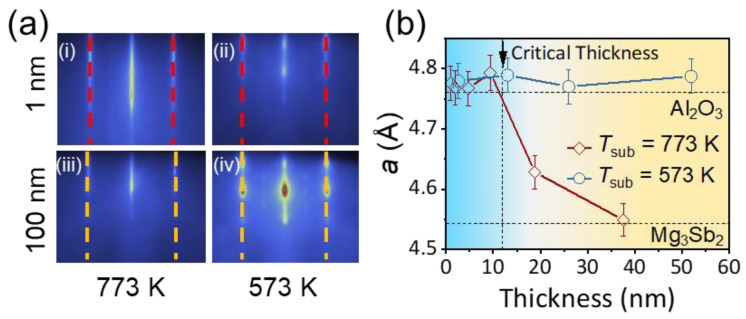
The evolution of in-plane lattice parameter with the deposition time for Mg_3_Sb_2_ films. (**a**) The evolution of RHEED patterns with thickness along the [10$\bar{1}$0] azimuthal direction of films. i. grown at *T*_sub_ = 773 K with thickness of 1 nm; ii. grown at *T*_sub_ = 573 K with thickness of 1 nm; iii. grown at *T*_sub_ = 773 K with thickness of 100 nm and iv. grown at *T*_sub_ = 573 K with thickness of 100 nm. The unchanged interval of RHEED streaks indicates the full strain state in the film grown at *T*_sub_ = 573 K. (**b**) The in-plane lattice parameter *a* as a function of film thickness for films grown at *T*_sub_ = 773 K and 573 K, quantified by the in situ RHEED measurements.

Figure 3 depicts the in situ RHEED patterns and the calculated in-plane lattice parameters *a* as a function of film thickness for our Mg_3_Sb_2_ films. As for the film grown at 773 K, the spacing of RHEED patterns (labeled by dashed lines) remains the same as that of the Al_2_O_3_ substrate when the film thickness is around 1 nm, indicating a tensile strained state. In addition, the spacing of RHEED patterns of the 100 nm thick film expands obviously as compared to the Al_2_O_3_ substrate, which reflects the release of lattice strain during the film growth. On the contrary, the RHEED streaks remain unchanged with film thickness of 1 nm and 100 nm for the Mg_3_Sb_2_ film grown at *T*_sub_ = 573 K, indicating this Mg_3_Sb_2_ film is in a full-strain state. Through the quantitative analysis of the in-situ RHEED patterns, we are able to clearly elucidate the evolution of *a* during the growth as well as to determine the critical film thickness *h*_c_ above which the strain relaxation will occur. It is remarkable that a high *T*_sub_ is crucial for releasing the epitaxial strain in Mg_3_Sb_2_, and the large epitaxial strain could remain in the thick film when the *T*_sub_ is very low. The tensile epitaxial strain is retained even up to 70 nm for the Mg_3_Sb_2_ film grown at *T*_sub_ = 573 K, as highlighted by the unchanged *a* value across the entire thickness. In the film grown at *T*_sub_ = 773 K, the *a* rapidly decays from *a* = 4.77 Å (the same as in the Al_2_O_3_ substrate) to *a* = 4.56 Å (identical to the value in the pristine Mg_3_Sb_2_) as the film thickness exceeds the *h*_c_, which is about 7 nm in this work. This phenomenon indicates the strain release process is related to the growth transition of the SK mode. 

The change of strain state in Mg_3_Sb_2_ films grown at different *T*_sub_ could be interpreted by the semi-empirical model of metastable layers proposed by Dodson and Tsao [33,34,35,36]. The migration of misfit dislocation could be thermally activated, and dominates the strain relaxation process in mismatched hetero-epitaxial films. The density of misfit dislocations could be qualitatively estimated by the FWHM of the Mg_3_Sb_2_ (0002) rocking curve as shown in Figure 1c: $\rho_{s}=\beta_{\left(002\right)}{}^{2}/\left(2\pi \ln2\times \left|b_{c}\right|\right)$, where β is the FWHM of the rocking curve, $\left|b_{c}\right|$ is the Burgers vector lengths equated to c-axial lattice constants and $\rho_{s}$ stands for the density of misfit dislocations, respectively [37,38,39,40]. The film grown at *T*_sub_ = 573 K shows a very high $\rho_{s}$ of ~4.88 × 10^14^ cm^−2^ while the $\rho_{s}$ strikingly decreases to 1.92 × 10^12^ cm^−2^ in the Mg_3_Sb_2_ film grown and annealed at *T*_sub_ = 773 K. Hence, the thermal process, such as a high *T*_sub_ and annealing, could effectively promote the dislocation migration and is beneficial for the relaxation of epitaxial strains in Mg_3_Sb_2_ films. Generally, epitaxial films of semiconductors such as Si_1-x_Ge_x_ [41], Ga_1-x_In_x_As [42] and Ga_1-x_Al_x_N [43] along with multiferroic Sr_2_IrO_4_ [44], SrRuO_3_ [45] and HoMnO_3_ [46] exhibit moderate biaxial epitaxial strains *ε* in the range of 0~4%. The prominent strain in the grown Mg_3_Sb_2_ films is closely related to the chemical bonding characteristics of Mg_3_Sb_2_, including weak bonds and a nearly isotropic 3D bonding network. The above study shows the feasibility of strain manipulation in Mg_3_Sb_2_-based compounds utilizing the strong interaction at the film-substrate interface and the decisive role of *T*_sub_. 

### 3.3. Detection and Manipulation of the Twin Structure

**Figure 4:**
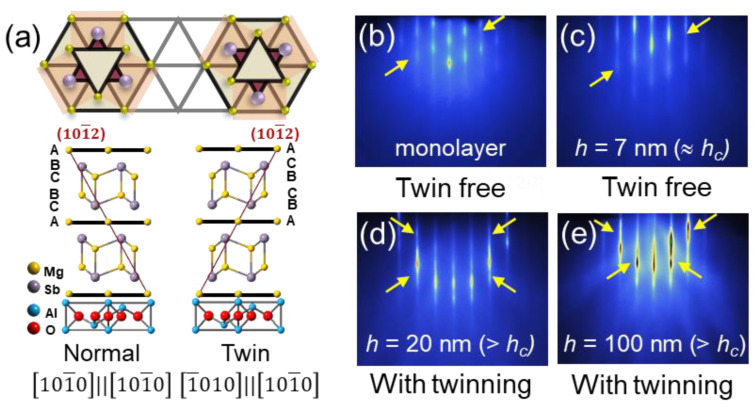
(**a**) Schematic stacking order of the twin structure in Mg_3_Sb_2_. Top and middle panels: two representative stacking orders in Mg_3_Sb_2_, where the 3-fold symmetry is visible in the top view. Bottom: the interlayer stacking follows a relation of [10$\bar{1}$0]//[10$\bar{1}$ 0] for the normal domain (left), while the stacking relation of [$\bar{1}$ 010]//[10$\bar{1}$ 0] represents the twin domain (right). The {$10\bar{1}2$} planes (in red) and the atomic stacking sequence (ABC-BCA and ACB-CBA) are drawn as references. (**b**–**e**) RHEED patterns measured during the deposition of the Mg_3_Sb_2_ film that is grown at *T*_sub_ = 773 K. The intensity modulation highlighted by arrows reveals the presence/absence of the twin structure. The mirror symmetric diffraction pattern indicates the existence of twin structure, while the diagonal feature depicts the absence of twin structure. (**b**,**c**) indicate the film with twin-free structure at the initial stage of deposition, whereas (**d**,**e**) indicate the twinning structure gradually forms when the film thickness during the further deposition.

Along with the strain evolution with film thickness and *T*_sub_, a 60° rotational twin structure is discovered in the Mg_3_Sb_2_ films, which could be efficiently regulated by tuning the thermal process. Measurements of RHEED and high resolution XRD are utilized to trace the formation and to identify the tuning mechanism of the twin structure in Mg_3_Sb_2_ films. As illustrated in Figure 4a, the normal domains and the twin domains differ in atomic stacking orders of ABC-BCA and ACB-CBA, respectively. In the RHEED patterns along the [11$\bar{2}$0] azimuthal direction, the diagonal feature of intensity modulation corresponds to the twin-free structure, while the mirror-symmetric RHEED pattern signifies the presence of twin structure (see Figure S3 and Figure 4b,c) [47]. For all of the displayed RHEED patterns, the symmetry feature is highlighted with arrows to reveal the presence/absence of the twin structure. Remarkably, in the Mg_3_Sb_2_ film grown at *T*_sub_ = 773 K, the RHEED patterns show a diagonal intensity modulation when the film thickness is below the *h_c_*, indicating a single domain structure. When the film thickness exceeds the *h_c_*, the twin structure forms rapidly so that the RHEED patterns become mirror symmetric (see Figure 4d,e). 

**Figure 5:**
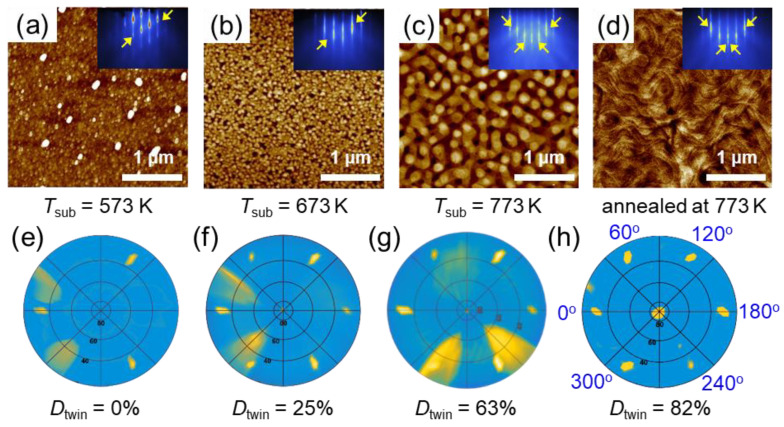
(**a**–**d**) AFM surface morphologies of the Mg_3_Sb_2_ films grown at various *T*_sub_. The inserted figures show the corresponding RHEED patterns along the [11$\bar{2}$0] azimuthal direction, where the intensity modulations are highlighted by yellow arrows. (**e**–**h**) XRD pole figures of the corresponding films shown in (**a**–**d**). The diffraction spots of normal domains are shown at 0°, 120° and 240°, while the spots of twin domains are evident at 60°, 180° and 300°. The *D*_twin_ denotes the degree of twinning that is calculated by Rigaku smartlab software based on the intensity and area of diffraction spots.

Figure 5 shows the twin domain formation with increasing the *T*_sub_ for Mg_3_Sb_2_ films. The film grown at *T*_sub_ = 573 K exhibits a macroscopic single-domain characteristic, yielding 3-fold symmetric diffraction spots at 0°, 120° and 240° in the pole figure, as shown in Figure 5e. In comparison, under a higher *T*_sub_ and by applying an annealing process, the twin structure forms in Mg_3_Sb_2_ films and its diffraction spots appear at 60°, 180° and 300° (see Figure 5f–h). Our results indicate that the content of twinning *D*_twin_ increases drastically with increasing the *T*_sub_, which is 25%, 63% and 82% for films grown at *T*_sub_ = 673 K and 773 K as well at *T*_sub_ = 773 K and in situ annealed, respectively. The twin structure is closely related to the strain relaxation process, and its formation is obviously promoted by elevating the *T*_sub_ and by annealing. Hence, thermal processes induce the improved mass transfer and the much reduced density of misfit dislocations is likely the mechanism responsible for the formation of the twin structure. The microscopic evidence of thermally promoted mass transfer is given by AFM and in situ STM measurements as shown in Figure 5a–d, Figures S4 and S5. On the one hand, the gain size increases greatly with increasing the *T*_sub_ in Mg_3_Sb_2_ films, e.g., from tens of nanometers to sub-micrometer with increase of the *T*_sub_ from 573 K to 773 K. On the other hand, annealing at 773 K not only further increases the grain size, but also significantly improves the crystallinity of the Mg_3_Sb_2_ film, forming a distinct layer structure with a flat surface. The Mg_3_Sb_2_ film grown at 773 K and annealed at 773 K for 20 min exhibits the most prominent layered structure with layer thickness of ~0.7 nm that is identical to the value of the single lattice spacing. 

### 3.4. Band Structure and the Tuning of E_F_

**Figure 6:**
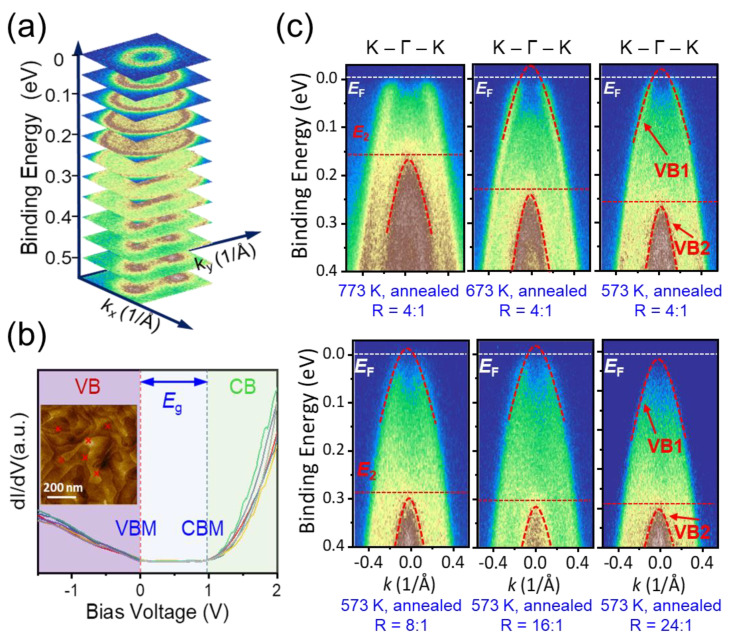
(**a**) ARPES maps of constant energy contours at different binding energies for the Mg_3_Sb_2_ film grown and in-situ annealed at *T*_sub_ = 773 K. (**b**) STS spectra of the Mg_3_Sb_2_ film in (**a**), which are measured from different regions (see the marks in the insert). The VBM, CBM and *E*_g_ indicate the valence band maximum, conduction band minimum and band gap, respectively. (**c**) ARPES spectra along the K-Γ-K direction of Mg_3_Sb_2_ films grown under different *T*_sub_ and Mg/Sb ratios R. The *E*_F_, VB1 and VB2 represent the Fermi energy, the topmost valence band, and the second valence band, respectively. Lowering the *T*_sub_ and increasing the R both shift the *E*_F_ towards the conduction band. *E*_2_ indicates the energy position of the VB2.

The characteristics of band structure and the tuning mechanism of *E*_F_ are crucial for the regulation and optimization of electronic properties [48,49,50,51,52]. Here, utilizing the single crystalline films grown by MBE, we are able to determine the band features of Mg_3_Sb_2_ by ARPES and STS measurements. Figure 6a,b depict the Fermi maps of constant energy contour at different binding energies *E*_b_ and the STS spectra of the Mg_3_Sb_2_ film grown at 773 K. The momentum dispersion of Fermi surfaces expands with increase of the *E*_b_, implying their valence band feature and *p*-type conduction of the grown Mg_3_Sb_2_ film. In addition, we observe a circular hole pocket centered at the Γ point and a single band feature near the *E*_F_, which evolves into a complex band configuration composed of two valence bands (denoted as VB1 and VB2). The VB2 appears at the *E*_b_ of ~0.25 eV. The STS measurements in different regions unambiguously validate the electronic density of states (DOS) versus bias voltage. The spectra indicate a band gap of *E*_g_ ≈ 0.89 eV, coinciding with the result from Mg_3_Sb_2_ bulks [53]. The *E*_F_ is located near the valence band maximum (VBM), corresponding to the ARPES result. The DOS near the conduction band maximum (CBM) is much higher than that near the VBM, in agreement with the higher DOS effective mass and superior electrical properties of n-type Mg_3_Sb_2_ compared to that of the *p*-type counterpart [5]. 

Figure 6c shows the energy-momentum dispersion along the K-Γ-K direction of Mg_3_Sb_2_ films grown at different *T*_sub_ and R. The growth parameters significantly alter the *E*_F_ of Mg_3_Sb_2_ films. The VBM is fully exposed in the film grown at *T*_sub_ = 573 K and R = 24. Obviously, the VBM of Mg_3_Sb_2_ is featured with two bands (VB1 and VB2) that show an energy interval of 0.3 eV, which is theoretically interpreted by the effect of crystal field energy splitting [15]. Since the saturated vapor pressure of the Mg element is two orders of magnitude higher than that of the Sb element under the adopted *T*_sub_ (573 K–773 K) [54], the growth of Mg_3_Sb_2_ films was always performed under a Mg-deficient condition. Since Mg vacancies ($V_{Mg}^{2-}$) possess the lowest formation energies among all point defects according to previous results [48], the formation of $V_{Mg}^{2-}$ is energetically favorable during the growth of Mg_3_Sb_2_ films. As a result, the acceptor defects $V_{Mg}^{2-}$ would push the *E*_F_ deeply into the VB, and result in a strong p-type conduction of Mg_3_Sb_2_ films. The Mg_3_Sb_2_ film grown at 773 K and annealed shows the strongest p-type conduction among all films, indicated by the highest energy positions of VB2. With increase of the R (i.e., the Mg flux) from 4 to 24 and decreasing the *T*_sub_ from 773 K to 573 K, the formation of Mg vacancies is effectively suppressed, resulting in an obvious *E*_F_ upshift of 200 meV. According to Luttinger’s theorem [55], such a sizeable *E*_F_ shift corresponds to the carrier density being altered by two orders of magnitude. This study manifests that the change of MBE processing parameters is an effective approach to manipulate the Mg content and hence the *E*_F_ of the grown Mg_3_Sb_2_ film. 

## 4. Conclusions

In summary, high crystalline quality Mg_3_Sb_2_ films were successfully epitaxially grown on lattice-mismatched Al_2_O_3_ (000*l*) substrates by the MBE technique. The growth of Mg_3_Sb_2_ films showed a layer-to-island morphological evolution with increase of film thickness, and could be interpreted by the Stranski–Krastanov (SK) growth characteristic. Films grown and annealed at a high *T*_sub_ of 773 K possessed a flat surface and distinct layered structure, implying a strong mass transfer process at elevated temperatures. The strong interlayer interaction between the film and the substrate plays a vital role in the manipulation of epitaxial strains and the formation of twin structure in the grown Mg_3_Sb_2_ films. On the one hand, the Mg_3_Sb_2_ film grown at 573 K was in a full tensile strain state, and lacked the twin structure. On the other hand, the Mg_3_Sb_2_ film grown and annealed at 773 K was completely strain relaxed when the film thickness was above a critical value of ~7 nm, and contained obvious twin structures with a large *D*_twin_ of up to 82% in the ultimate case. This is ascribed to the effects from thermally activated mass transfer and the effective migration of misfit dislocations. The band structure characterizations confirmed an intrinsic p-type conduction of the grown Mg_3_Sb_2_ films, which is due to the Mg deficiency during the film growth and the formation of donor-like Mg vacancies. Moreover, decreasing the growth temperature and increasing the Mg/Sb flux ratio could remarkably enhance the Mg content in the grown films and suppress the formation of Mg vacancies, leading to an obvious Fermi level downshift of ~0.2 eV. This work elucidated the tuning effects regarding epitaxial strains, twin structure, film crystallinity and Fermi level of Mg_3_Sb_2_ films, and thus laid an important foundation for rationally manipulating their microstructure, electronic band structure and thermoelectric performance. 

## Data Availability

Data available on request from authors.

## Supplementary Materials

The following supporting information can be downloaded at: https://www.mdpi.com/article/10.3390/nano12244429/s1, Figure S1: The RHEED patterns of Mg_3_Sb_2_ films grown on Al_2_O_3_ (000*l*) substrates with and without passivation treatment; Figure S2: The RHEED patterns of Mg_3_Sb_2_ grown on different substrates; Figure S3: Schematic illustration of the RHEED measurement and the RHEED patterns for twin-free structure and twin domains; Figure S4: STM surface morphology and structure evolution of Mg_3_Sb_2_ films grown at 773 K; Figure S5: Layer structure of the Mg_3_Sb_2_ film grown at 773 K and annealed at 773 K for 20 min.
