# Supplementary material for: Temperature-Driven Twin Structure Formation and Electronic Structure of Epitaxially Grown Mg3Sb2 Films on Mismatched Substrates
Source: Nanomaterials (Basel). 2022 Dec 12;12(24):4429. doi: 10.3390/nano12244429 (PMC9783249; doi:10.3390/nano12244429)
Supplement: Supplementary file 1 [file nanomaterials-12-04429-s001.zip › nanomaterials-2091552-supplementary.pdf]

Supporting information:

Temperature-Driven Twin Structure Formation and Electronic Structure of Epitaxially Grown Mg_3Sb_2 Films on Mismatched Substrates

Sen Xie [1,2,†], Yujie Ouyang [1,2,†], Wei Liu [1,*], Fan Yan [1], Jiangfan Luo [1], Xianda Li [1], Ziyu Wang [3], Yong Liu [4,*] and Xinfeng Tang [1,*]

[1] State Key Laboratory of Advanced Technology for Materials Synthesis and Processing, Wuhan University of Technology, Wuhan 430070, China
[2] International School of Materials Science and Engineering, Wuhan University of Technology, Wuhan 430070, China
[3] The Institute of Technological Sciences, Wuhan University, Wuhan 430072, China; zywang@whu.edu.cn
[4] School of Physics and Technology and The Key Laboratory of Artificial Micro- and Nano-Structures of Ministry of Education, Wuhan University, Wuhan 430072, China
* Correspondence: w.liu@whut.edu.cn (W.L.); yongliu@whu.edu.cn (Y.L.); tangxf@whut.edu.cn (X.T.)
† These authors contributed equally to this work.

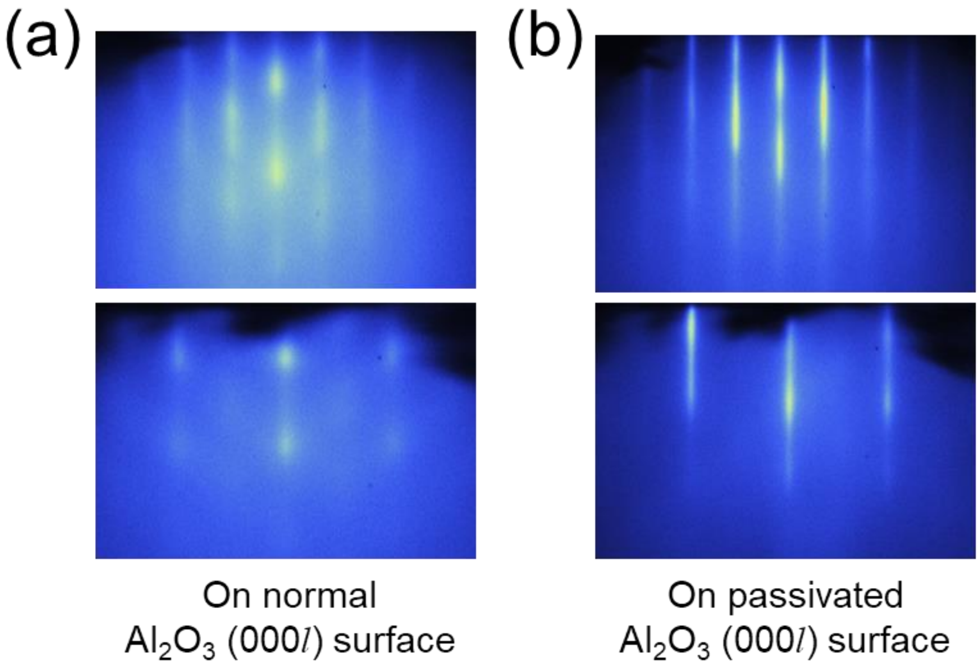

Figure S1. The RHEED patterns of Mg_3Sb_2 films grown on Al_2O_3 (000*l*) substrates with and without passivation treatment.

The RHEED patterns of Mg3Sb2 grown on different substrates

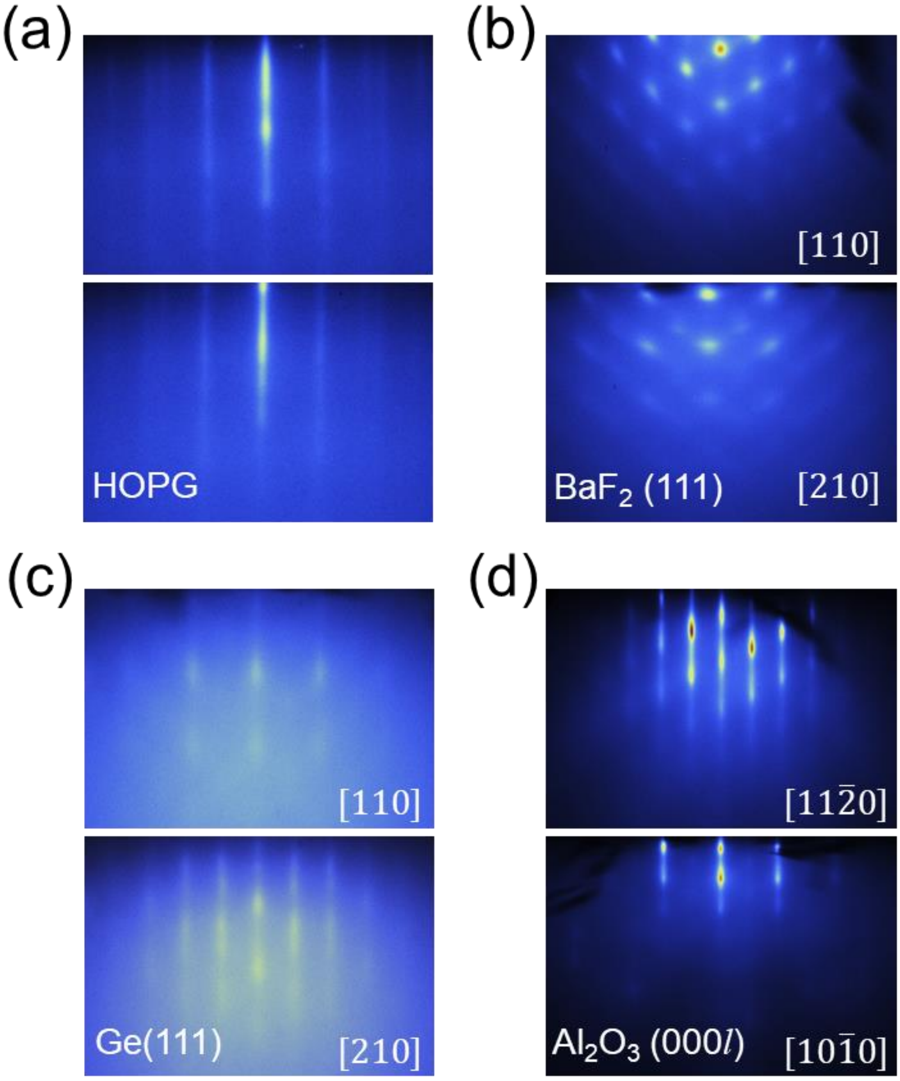

Figure S2. The RHEED patterns of Mg_3Sb_2 grown on different substrates: (**a**) cleaved HOPG, (**b**) BaF_2 (111), (**c**) Ge (111), and (**d**) Al_2O_3 (000*l*) substrates. The Mg_3Sb_2 grown on Al_2O_3 shows the highest crystallinity.

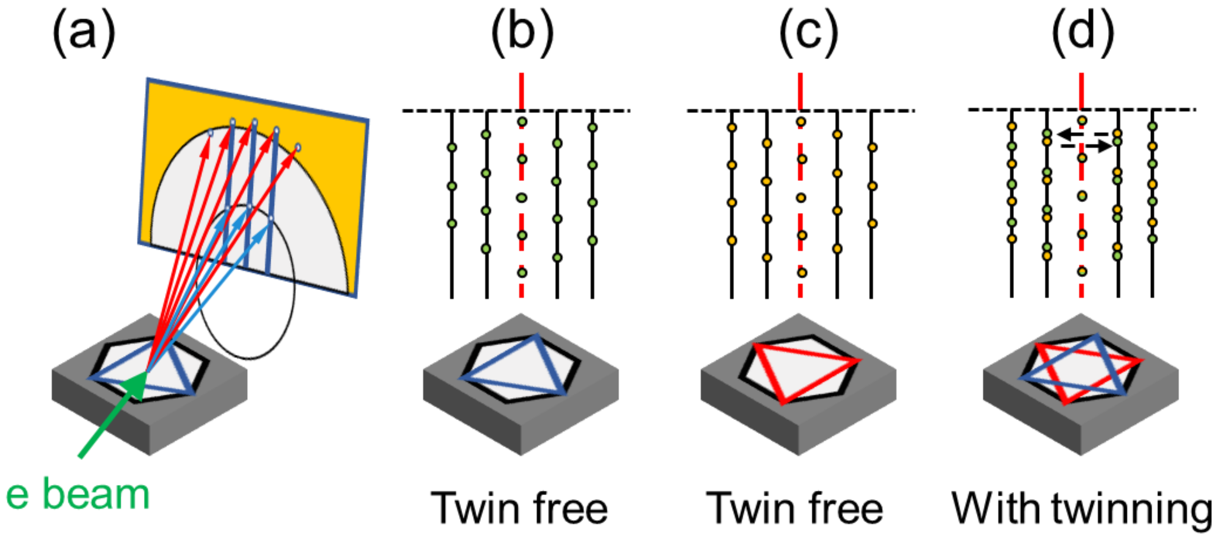

Figure S3. (**a**) Schematic illustration of the RHEED measurement. (**b-d**) RHHED patterns of hexagonal lattice with and without twin domains.

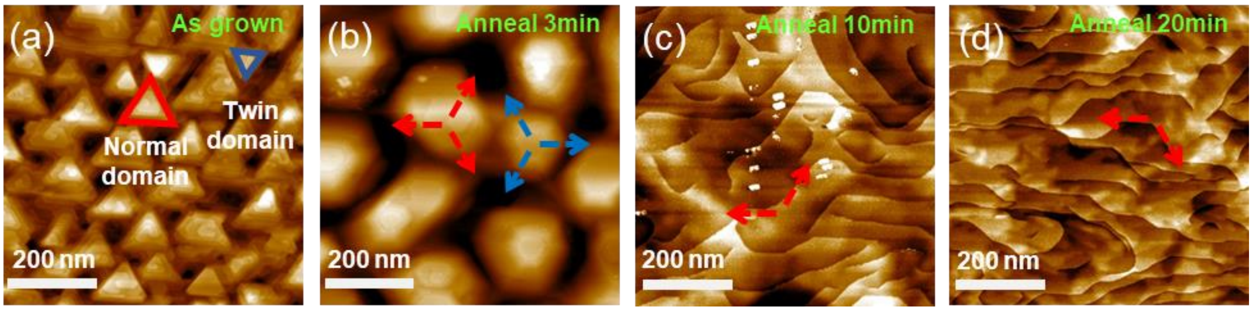

Figure S4. STM surface morphology and structure evolution of Mg_3Sb_2 films grown at 773 K: **(a)**, as-grown; **(b)**, annealed for 3 mins; **(c)**, annealed for 10 mins; **(d)**, annealed for 20 mins. Twin domains are marked by blue triangles in (a) and blue arrows in (b). Annealing at 773 K significantly promotes the crystallinity of films, which is evident from the prominent layer structure and increased grain size.

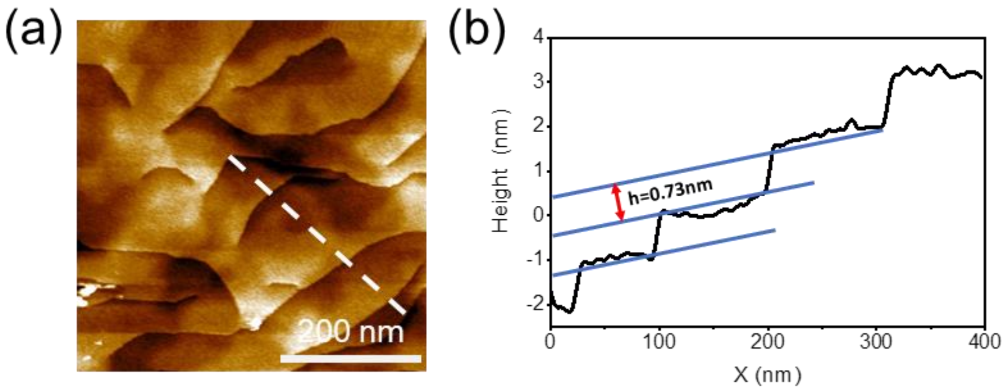

Figure S5. Layer structure of the Mg_3Sb_2 film grown at 773 K and annealed at 773 K for 20 mins: (a) STM surface morphology, (b) The height profile along the white dashed line in (a). The thickness of a single layer is ~ 0.7 nm, in agreement with the spacing of a single lattice layer.
